# Genome-wide identification and expression analysis of the anthocyanin-related genes during seed coat development in six *Brassica* species

**DOI:** 10.1186/s12864-023-09170-2

**Published:** 2023-03-09

**Authors:** Daozong Chen, Haidong Chen, Guoqiang Dai, Haimei Zhang, Yi Liu, Wenjie Shen, Bo Zhu, Cheng Cui, Chen Tan

**Affiliations:** 1 College of Life Sciences, Ganzhou Key Laboratory of Greenhouse Vegetable, Gannan Normal University, Ganzhou, 341000 China; 2 Crop Research Institute, Sichuan Academy of Agricultural Sciences, Chendu, 610066 China

**Keywords:** *Brassica*, Seed coat color, Anthocyanin-related genes, *MYB5*, *TT2*

## Abstract

Yellow seed is one favorite trait for the breeding of *Brassica* oilseed crops, but the performance of seed coat color is very complicated due to the involvement of various pigments. The change of seed coat color of *Brassica* crops is related to the specific synthesis and accumulation of anthocyanin, and the expression level of structural genes in anthocyanin synthesis pathway is specifically regulated by transcription factors. Despite some previous reports on the regulations of seed coat color from linkage marker development, gene fine-mapping and multi-omics association analysis, the trait of *Brassica* crops is affected by the evolutionary events such as genome triploidization, the regulatory mechanism is still largely unknown. In this study, we identified genes related to anthocyanin synthesis in six *Brassica* crops in U-triangle at the genome-wide level and performed collinearity analysis. A total of 1119 anthocyanin-related genes were identified, the collinear relationship of anthocyanin-related genes on subgenomic chromosomes was the best in *B. napus* (AACC) and the worst in *B. carinata* (BBCC). The comparisons of gene expressions for anthocyanin metabolic pathways in seed coats during seed development revealed differences in its metabolism among these species. Interestingly, the R2R3-MYB transcription factors *MYB5* and *TT2* were differentially expressed at all eight stages of seed coat development, indicating that they might be the key genes that caused the variation of the seed coat color. The expression curve and trend analyses of the seed coat development period showed that the main reason for the unexpressed copies of *MYB5* and *TT2* was likely gene silencing caused by gene structural variation. These results were valuable for the genetic improvement of *Brassica* seed coat color, and also provided new insights into gene multicopy evolution in *Brassica* polyploids.

**Supplementary Information:**

The online version contains supplementary material available at 10.1186/s12864-023-09170-2.

## Introduction

The *Brassica* species in U's triangle include three diploid crops, *B. rapa* (AA, 2n = 20), *B. nigra* (BB, 2n = 16), *B. oleracea* (CC, 2n = 18) and three allotetraploid crops, *B. juncea* (AABB, 2n = 36), *B. napus* (AACC, 2n = 38), *B. carinata* (BBCC, 2n = 34), formed by natural pairwise hybridizations of three diploids (U, 1935). The *Brassica* species comprise the important oil crops and vegetables and are widely cultivated worldwide for vegetable and oil acquisition. Generally, the seeds of these *Brassica* species vary in color from yellow, brown to dark brown or black [45, 69] Previously, yellow seed was a desirable /+trait with great potential to improve the quality of seed oil in *Brassica* crops, because yellow seeds have the thinner coats, a reduced percentage of pigment and hull, and a greater content of oil and protein than the black seeds [68, 38, 40, 56]. In recent years, with these superior characteristics, the yellow seed coat trait of *B. rapa* [61, 81, 82], *B. napus* [20, 21, 29, 30, 79, 86] and *B. juncea* [50, 64] has become the focus of global rapeseed breeding.

In nature, varieties with different seed colors have different seed coat pigment components. In *Arabidopsis* and *Brassica* crops, kaempferol derivatives, quercetin derivatives, isorhamnetin derivatives and epicatechin glycosides are the main pigments of seeds [31, 61, 62, 64, 79]. Importantly, the differences in the content of flavonoids, especially proanthocyanidins (PAs), are the main substances that cause color variation of seed coat color [27, 46, 55, 79, 82]. In the model plant *Arabidopsis thaliana*, the biosynthetic pathway of anthocyanins has been clearly reported. From phenylpropane synthesis pathway (*PAL*, *C4H*, *4CL*), flavonoid synthesis pathway (*CHS*, *CHI*, *F3H*/*F3’H*/*F3′5’H*) to anthocyanin synthesis pathway (*DFR*, *ANS*, *UFGT*, etc.), these series of synthases are regulated by three types of genes: MYB transcription factor, bHLH transcription factor and WD-40 protein [53, 76]. In addition, the MBW transcriptional regulatory complexes TT2-TT8-TTG1, MYB5-TT8-TTG1, TT2-EGL3-TTG1, and TT2-GL3-TTG1, composed of three types of transcription factors, were reported to be involved in the synthesis of anthocyanins in the seed coat [76].

For decades, genes that control seed coat color have been studied in different *Brassica* species cultivars collected around the world, by exploiting QTL mapping, resequencing analysis, transcriptome analysis, transcriptome combined metabolome analysis, etc. Numerous studies have revealed that the seed color of *Arabidopsis* and *Brassica* crops was controlled by few major QTLs [50, 57, 74, 76]. Among them, several homologs to *Arabidopsis* transcriptional regulators affecting seed color, such as *MYB11*, *MYB12*, *MYB111*, *TT2*, *TT8*, *GL3*, *EGL3* have been identified, and some of them have been functionally validated in *B. rapa*, *B. juncea* and *B. napus* [64, 79, 82]. Nevertheless, due to differences in genetic background of materials and lack of systematic data, the exact underlying regulatory mechanism of seed coat color remains unclear [28, 50, 64, 72, 75, 79, 80, 82].

In this study, we identified genes related to anthocyanin synthesis in *Brassica* crops at the genome-wide level and performed collinearity analysis. In addition, we collected the transcriptome sequencing data of the seed coats at eight stages of seed development in six *Brassica* species, and we analyzed the anthocyanin-related differentially expressed genes and their expression levels in different developmental stages of the seed coat. Additionally, *MYB5* and *TT2* were differentially expressed at all eight stages of seed coats in the six *Brassica* species. Our findings lay a foundation for elucidating the regulatory mechanisms of seed coat color in *Brassica* species and provide important resources for a comparative and integrated analysis of seed coat color trait in these interrelated *Brassica* species.

## Materials and methods

### Identification of anthocyanin-related genes in *Brassica* species

In this study, the genome and protein sequences of the *B. rapa* (Chiifu-401–42 v3.0), *B. oleracea* (HDEM), *B. nigra* (Ni100-LR), *B. napus* (Darmor*-bzh* v10) were downloaded from the BRAD database [7]; http://brassicadb.cn), *B. juncea* (SCYZ) genome sequence from NCBI PRJNA615316 [22] (https://www.ncbi.nlm.nih.gov/), *B. carinata* (zd-1) genome sequence from GenBank JAAMPC000000000 [66] (https://www.ncbi.nlm.nih.gov/), and the anthocyanin-related genes genome and protein sequences were downloaded from the *Arabidopsis* database (TAIR; http://www.arabidopsis.org/index.jsp). In order to accurately identify anthocyanin-related genes, we mainly divide it into the following steps: Firstly, local BLASTP has been used to search anthocyanin-related genes with E-value < 1e-20, 55 anthocyanin-related genes protein sequences were derived from Arabidopsis. Secondly, the candidate anthocyanin-related genes in the six *Brassica* species were identified by a local BLASTN search with 55 anthocyanin-related genes coding sequence from *Arabidopsis* to identify candidates with E-value < 1e-20, identity > 70%, coverage > 60%. Thirdly, SynOrths software [8] has been used to determining the collinear orthologous of two genes based on their own sequence similarity and the homology of their flanking genes, and then extracting the colinear genes of anthocyanin-related genes. Finally, the BLASTP, BLASTN and SynOrths software identified results were pooled and deduplicated, and determined in conjunction with PFAM protein family database (https://pfam.xfam.org/).

### Chromosomal location and synteny analysis

The genome annotation data were collected and mapped on the chromosomes using the TBtools software (v0.67) to identify the physical chromosomal location of all anthocyanin-related genes in *Arabidopsis* and six *Brassica* species [4]. The collinearity of intraspecific and interspecific genes was determined using the BLASTP (E-value: 1e-10, max_target_seqs:1) and Multiple Collinearity Scan toolkit (MCSscanX, gap_penalty: -1, E-value: 1e-10) [71], SynOrths software (E-value < 1e-20, Query gene = 20, Reference gene = 100) has been used to determining the collinear orthologous [8], TBtools software (v0.67) was used to drop the collinearity genes on each chromosome [4].

### Expression profiles analysis based on RNA-seq data

The raw data of 144 seed coats RNA-seq data of six *Brassica* species, *B. rapa* (Parkland-R), *B. oleracea* (Chinese Kale-O), *B. nigra* (CR2748-N), *B. napus* (DH12075-P), *B. juncea* (AC Vulcan-J), *B. carinata* (C901163-C) with eight developmental stages (Unfertilized ovule integuments (UO; no embryo), 1- to 2-cell zygote stage (S1), 4- to 8-cell stage (S2, 8-cell stage shown), 16- to 64-cell stage (S3, globular stage shown), heart stage(S4), torpedo stage(S5), bent stage(S6), and mature (S7) stage of seed formation) were collected from Gene Expression Omnibus under accession no. GSE153257. Low-quality reads were removed from the raw reads using Cutadapt and Trimmomatic software to get clean reads [39, 2]. Clean reads were mapped to the corresponding reference genome using HISAT2 software [51]. Gene expression levels of each gene were calculated using StringTie and Ballgown software [51]. The read counts of each gene were calculated using the htseq-count function in htseq software [1]. The R package DEseq2 (v1.16.1) was used to identify the differentially expressed genes (DEGs) between leaves of different colors based on the following criteria: padj < 0.05 & log2FoldChange > 2 [5].

### Co-differential expression analysis of anthocyanin-related genes

The RNA-seq data were used to perform co-expression network analysis using R language (v4.2.1). In order to calculate the adjacent order function formed by the gene network and the difference coefficients of different nodes, the TOM similarity algorithm calculates the co-expression correlation matrix to express the gene correlation in the network. The correlation network diagram was drawn by extracting the non-weight coefficients (weight) of anthocyanin-related genes in the matrix. STRING software (https://version-11-5.string-db.org/) was used to reveal a co-expression plot [33, 70].

### Phylogenetic, promoter characteristics, gene structure, conserved motifs analysis of TT2 and MYB5

The *TT2* and *MYB5* protein sequences of the six *Brassica* species and *Arabidopsis* were used to generate phylogenetic trees via ClustalX [26] and MAFFT sofaware (Katoh and Standley, 2013) multiple sequence alignments with the default parameters. A maximum likelihood (ML) phylogenetic tree was constructed using FastTree2 software (v2.1.11), in which JTT (Jones-Taylor-Thornton) model was the best substitution model [52]. The *TT2* and *MYB5* promoter regions of 2000 bp regions upstream of the translational start sites ATG were examined based on their positions in the genomes of six *Brassica* species and *Arabidopsis* using Samtools software (v 1.8), which was used to identify the cis-elements in the promoters according to the online PlantCARE database (http://bioinformatics.psb.ugent.be/webtools/plantcare/html/). The gene structures of *TT2* and *MYB5* were analyzed according to the GFF annotation file of the gene position information in the six *Brassica* crops and *Arabidopsis* database. The MEME online tool (https://meme-suite.org/meme/) was used to investigate conserved domains, and the WEBLoGo online tool (https://weblogo.berkeley.edu/) and SWISS-MODEL online tool (https://swissmodel.expasy.org/) was used to draw spatial structure. TBtools software (v0.67) was used to draw the *TT2* and *MYB5* to the different copies of each *Brassica* species, including phylogenetic, promoter characteristics, gene structure, conserved motifs [4].

### Construction of expression profiles of TT2 and MYB5 during seed coat development based on RNA-seq data

Transcriptome data of all eight periods of seed coat development of six *Brassica* species were used to analyze the expression patterns and trends of *TT2* and *MYB5*. The R package ggplot2 (v3.3.6) was used to draw the expression pattern of *TT2* and *MYB5*, and R package ggridges (v0.5.3) was used to draw the expression trends of *TT2* and *MYB5*.

## Results

### Identification and characterization of anthocyanin-related genes

In order to comprehensively and accurately identify anthocyanin-related genes, the 55 anthocyanin-related genes protein sequences of *Arabidopsis* were downloaded and used as seed sequences to search the protein sequences database of *Arabidopsis* and six *Brassica* species to identify homologous anthocyanin-related genes in six *Brassica* crops. The BLASTP (protein to protein), BLASTN (CDS to CDS) and SynOrths software (protein to protein, orthologous gene identification) identified results were pooled and deduplicated, and then the orthologous and paralogous genes determined by PFAM. A total of 1119 anthocyanin-related genes were identified, these included 120 in *B. rapa*, 131 in *B. oleracea*, 135 in *B. nigra*, 258 in *B. napus*, 229 in *B. juncea* and 246 in *B. carinata* (Table 1, Supplementary Table S1). Among the three subgenomes of A, B, and C, the C subgenome had the most distribution and the A subgenome the least (Table 1).

**Table 1:** Anthocyanin-related genes distribution in U's triangle *Brassica* species

Species	Referenc_genome	Gene number				
		**AA**	**BB**	**CC**	**unknow**	**Total**
*B.rapa*	Chiifu3.0	120			0	120
*B.nigra*	Ni100-LR		135		0	135
*B.oleracea*	HDEM			131	0	131
*B.juncea*	SCYZ	105	123		1	229
*B.napus*	Darmor-bzh v10	116		141	1	258
*B.carinata*	Zd-1		99	122	24	246
total		341	357	394	26	1119

### Chromosomal distribution and collinearity analysis of anthocyanin-related genes in six *Brassica* species

**Fig. 1:**
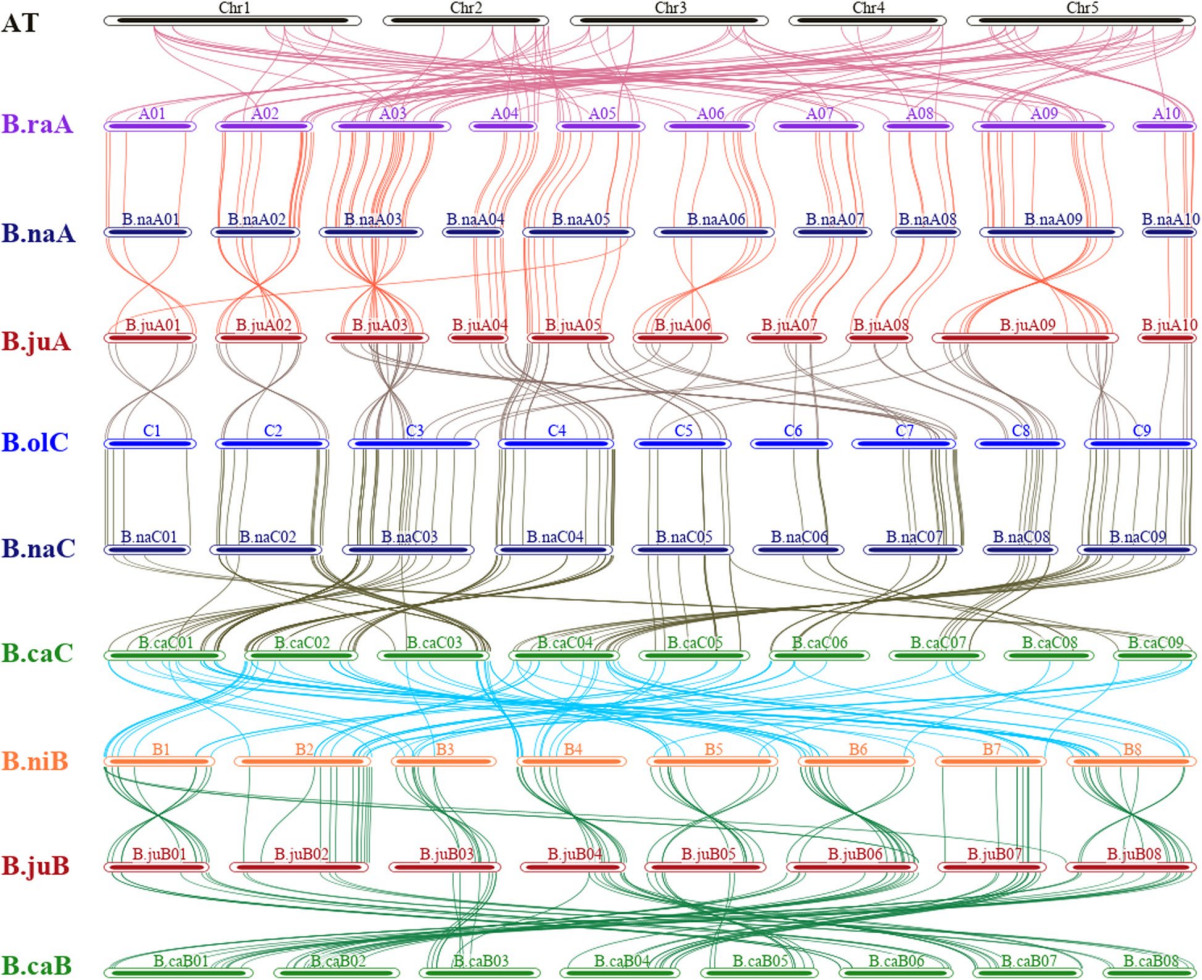
Interspecies synteny of anthocyanin-related genes between the six species in U’s triangle and *Arabidopsis*. anthocyanin-related genes colinearity of the genomes of the six *Brassica* species in U’s triangle and *Arabidopsis*, including three diploid species, *B. rapa* (A genome, BraA), *B. nigra* (B genome, BniB), and *B. oleracea* (C genome, BolC) and three tetraploid species, *B. napus* (AACC, BnaA, and BnaC subgenomes); *B. juncea* (AABB, BjuA and BjuB subgenomes); and *B. carinata* (BBCC, BcaB and BcaC subgenomes)

To better understand the copy number variation and collinearity of anthocyanins related genes, we mapped all 1174 genes to the genome chromosomes of 7 corresponding species. Chromosome mapping showed that they were located on all chromosomes of each species: 55 genes on 5 chromosomes of *Arabidopsis*, 120 genes on 10 chromosomes of *B. rapa*, 131 genes of *B. oleracea* on 9 chromosomes, 135 genes of *B. nigra* on 8 chromosomes, 258 genes of *B. napus* on 19 chromosomes, 229 genes of *B. juncea* on 18 chromosomes, and 246 genes of *B. carinata* on 17 chromosomes (Fig. 1). Chromosomal distributions showed that these genes were evenly distributed in the three subgenomes of the six *Brassica* crops, and the copy number of the tetraploids was about the sum of the corresponding subgenomes (Table 1).

The interspecific collinearity between *Arabidopsis thaliana* and six *Brassica* species was also analyzed to further explore the evolution of anthocyanin-related genes (Fig. 1). At the genome-wide level, there was a one-to-one collinear relationship between the copies of these genes in the A subgenomes from *B. rapa*_(BraA) and *B. napus*_(BnaA), *B. oleracea*_(BolC) and *B. napus*_(BnaC). Interestingly, in the subgenomes between *B. rapa*_(BraA) and *B. juncea*_(BjuA), *B. nigra*_(BniB) and *B. juncea*_(BjuB), in spite of the better collinearity, there were similar inversions between most chromosome arms. In addition, in the subgenomes between *B. nigra*_(BniB) and *B. carinata*_(BcaB), *B. oleracea*_(BolC) and *B. carinata*_(BcaC), the collinearity between different copies of the genes was poor, indicating that there might be a large number of rearrangement events at the chromosomal level after the formation of *B. carinata* (Fig. 1).

### Analysis of the transcriptome and anthocyanin-related gene expression patterns

**Fig. 2:**
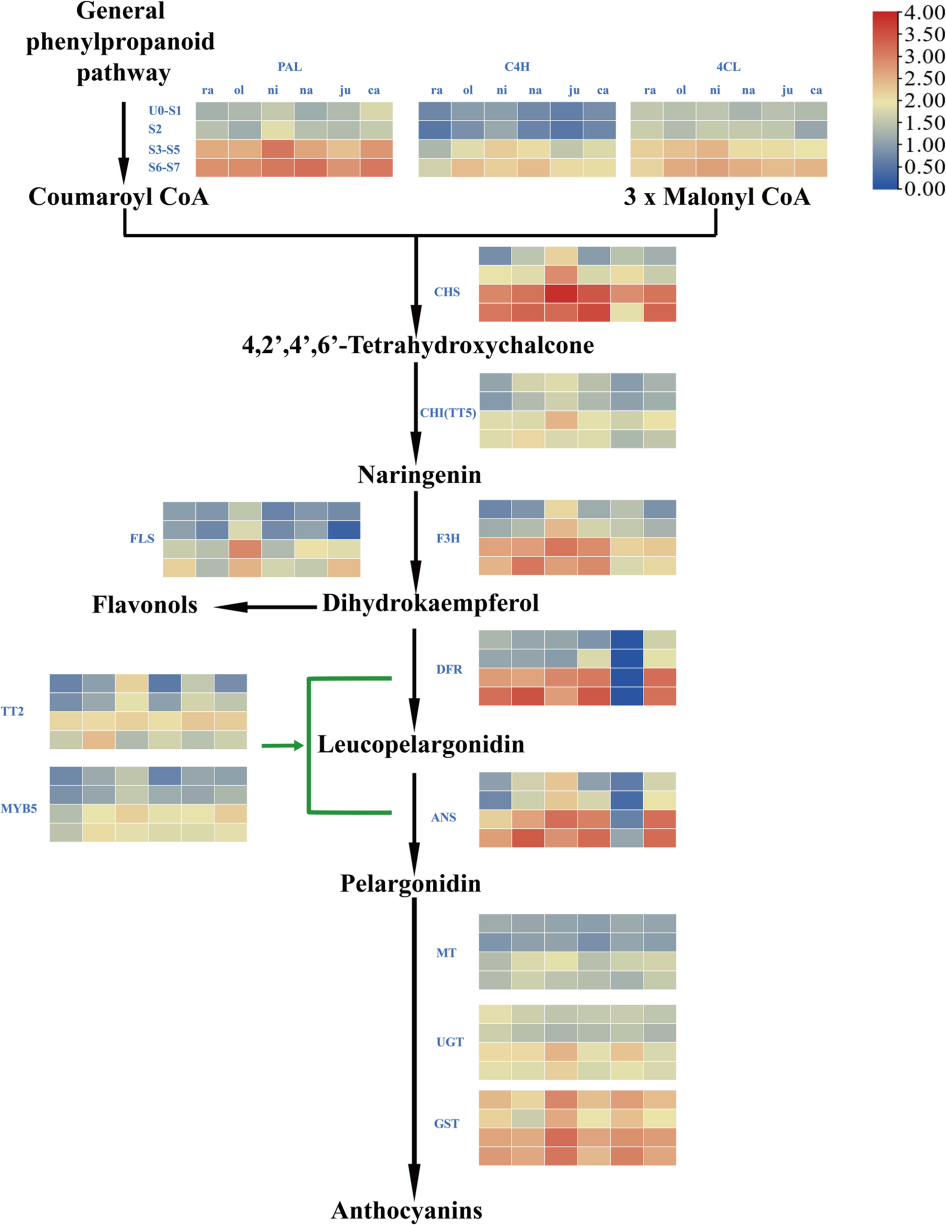
Anthocyanin-related gene expression patterns of six U's triangle *Brassica* species in eight periods of seed coat development

In order to explore the molecular mechanism of anthocyanin biosynthetic pathway-related genes regulating seed coat color formation during seed development, transcriptome data at eight periods (UO, S1, S2, S3, S4, S5, S6, S7) of seed coat development of six *Brassica* species were used to construct gene expression profiles (Fig. 2). In order to better show the expression patterns, the expression data of U0-S7 in a total of 8 developmental stages were divided into 4 stages, namely UO-S1, S2, S3-S5 and S6-S7 (Fig. 2). The results showed that the genes related to the anthocyanin synthesis pathway had high expression levels in all two stages, S3-S5 and S6-S7, which indicated that the synthesis of anthocyanins in the seed coat might mainly start from the S3 period (Fig. 2). In general, the expression levels of structural genes were higher than those of transcription factors *MYB5* and *TT2* in each developmental stage, especially in the S3-S7 developmental stage. Similar to previous research, glycosyltransferases (GST) were expressed at high levels in all developmental stages, indicating that glycosylation might be the main modification mode of anthocyanin color development. Interestingly, *DFR* and *ANS* (*LDOX*) genes were scarcely expressed in all developmental stages of *B. juncea*, which was quite different from the other five *Brassica* species.

**Fig. 3:**
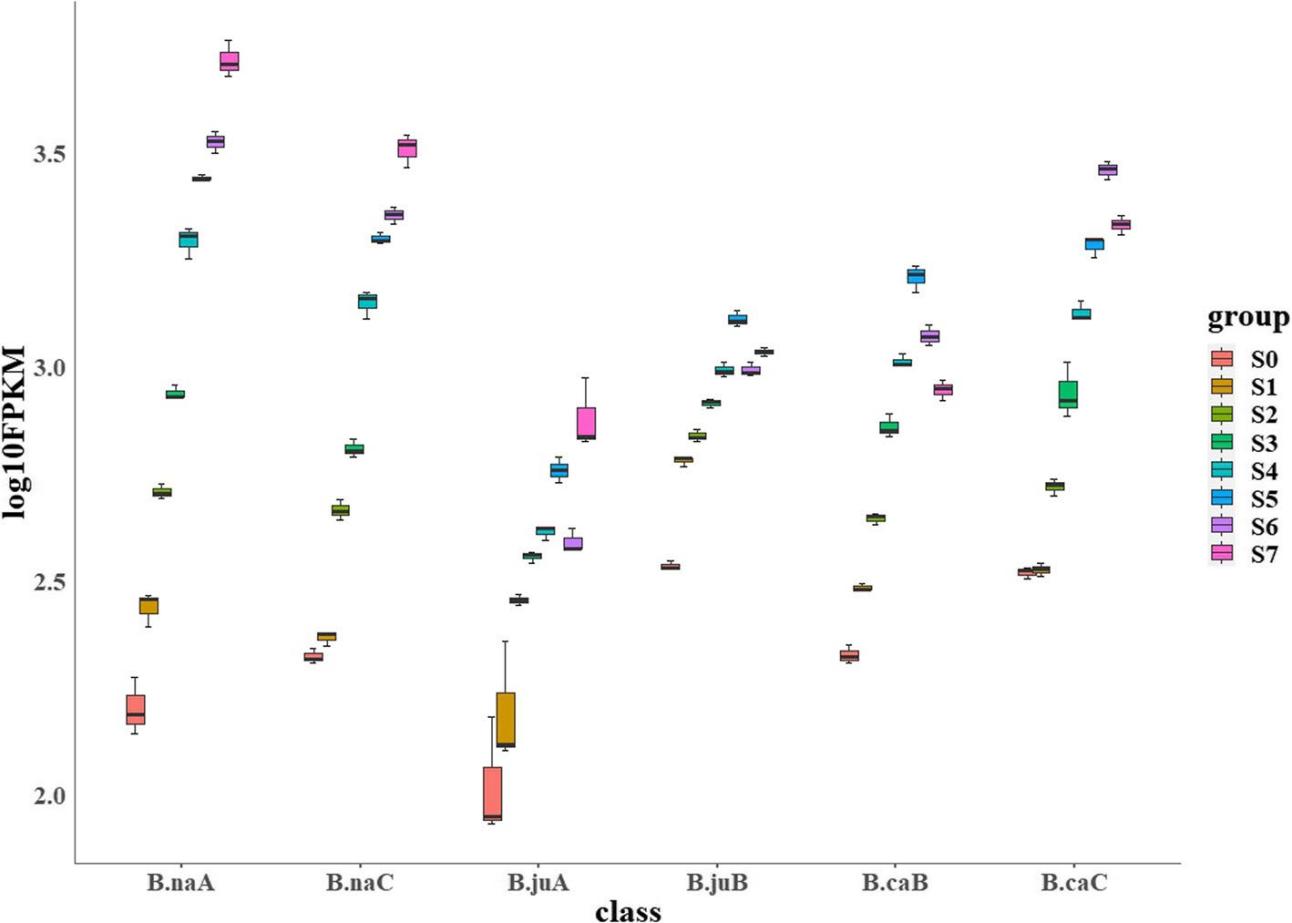
Phylogenetic, gene structural analysis and conserved motif identification of *MYB5* and *TT2*. *MYB5* and *TT2* mainly contain nine protein conserved domains, which are marked with different colors. Predicted cis-elements in *MYB5* and *TT2* promoters and gene structure. Promoter sequences (-2,000 bp) of *MYB5* and *TT2* were analyzed using PlantCARE, different shapes and colors represent different elements. Gene regions are marked with black lines and green modules, respectively, with black lines representing introns and green modules representing exons

Tetraploidy origin of *Brassica* crops brought abundant homologous gene copies. In order to understand how subgenome homoeologs were coordinately expressed during anthocyanin biosynthesis, we identified scenarios by comparisons among three tetraploid species. In general, the expression dominance of BnaA subgenome was significant over the BnaC subgenome in *B. napus*, the expression dominance of BjuB subgenome was obvious over the BjuA subgenome in *B. juncea*, but the expression dominance of BcaC subgenome was over the BcaB subgenome in *B. carinata* (Fig. 3). Unlike the pattern of expression bias at the subgenomic level (C > B > A) reported by Tan et al. [67], we did not find a significantly elevated number of dominant features in any subgenome in the three allotetraploid species (Fig. 3), suggesting a different transcriptional regulation of biased and dominant expression, which was consistent with the report by Gao et al. [13].

### Co-expression analysis of anthocyanin-related genes

**Fig. 4:**
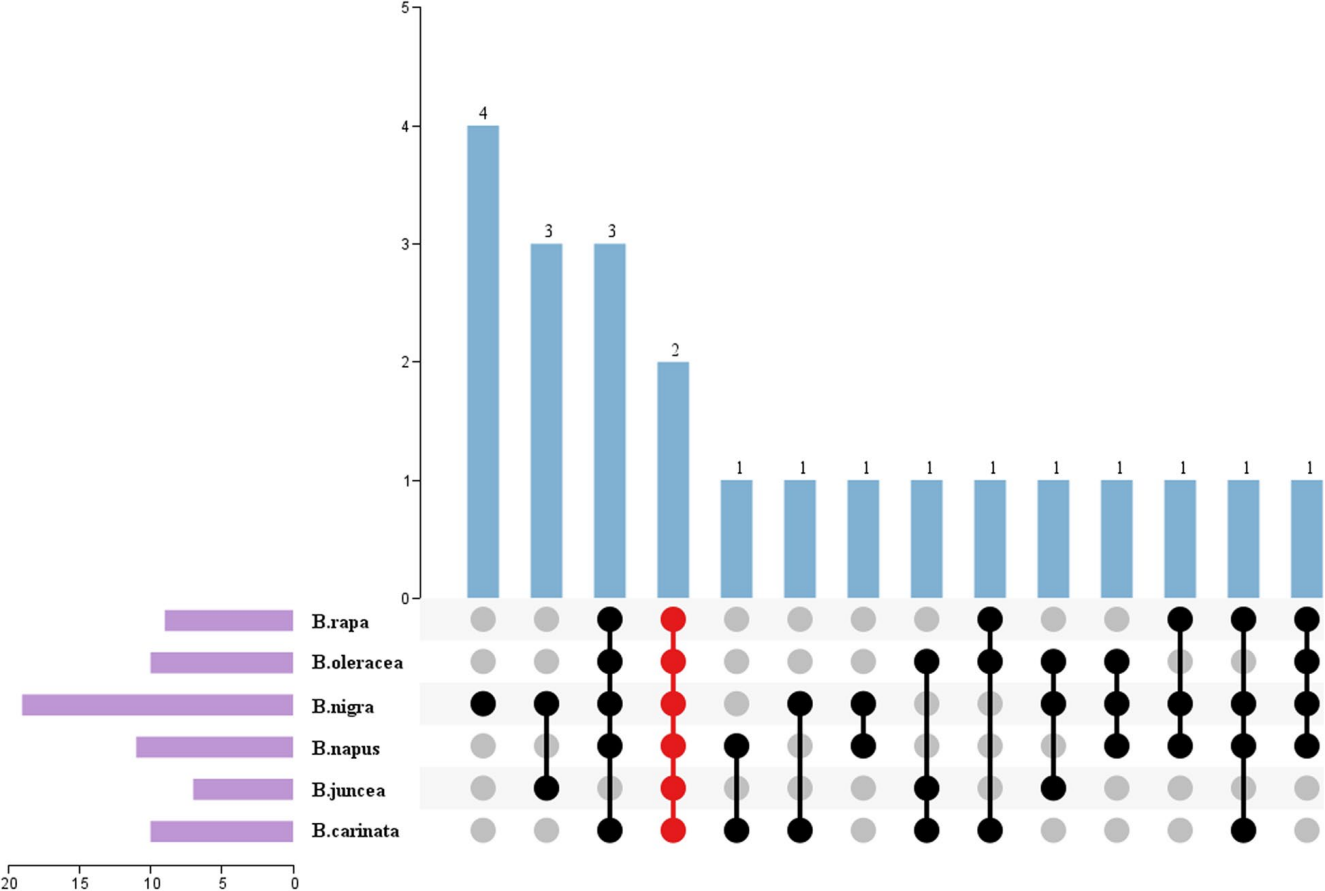
The differentially expressed anthocyanin-related genes between six *Brassica* crops seed coats of eight development RNA-seq data. The red color indicates the co-differentially expressed anthocyanin-related genes in six *Brassica* crops, the purple column indicated that anthocyanin related genes were differentially expressed in 8 developmental stages of the same material, the blue column shows the number of differentially expressed genes in common across all conditions

In order to identify the key genes that regulated the formation of seed coat color during seed coat development, the transcriptome data of seed coats at 8 stages of six *Brassica* species were used for differentially expressed gene (DEG) analysis. Among the anthocyanin-related DEGs, a total of 95 genes were differentially expressed in the transcriptome data of all eight developmental stages, with 9, 23, 13, 19, 12, 19 DEGs from *B. rapa*, *B. nigra, B. oleracea*, *B. napus*, *B. juncea* and *B. carinata*, respectively (Supplementary Table S2). Interestingly, only *MYB5* (*AT3G13540*) and *TT2* (*AT5G35550*) homologous copies were differentially expressed in all eight stages of six *Brassica* crops (Fig. 4, Supplementary Table S2). This was different from the results we discovered that only *PAP1/2* homologous copies were differentially expressed in green and purple leaves of five *Brassica* crops [5], indicating the different transcriptional regulations of anthocyanins between vegetative and reproductive organs in *Brassica* crops. At the same time, *DFR*(*AT5G42800*), *MYB5*(*AT3G13540*) and *AHA10*(*AT1G17260*) were differentially expressed in eight developmental stages of five *Brassica* crops, especially *DFR*(*AT5G42800*), which iwa generally considered to be the target gene of the MBW transcriptional regulatory complex, was not differentially expressed only in *B. juncea* (Fig. 4, Supplementary Table S2).

### Phylogenetics, gene structure and conserved motif of MYB5 and TT2

**Fig. 5:**
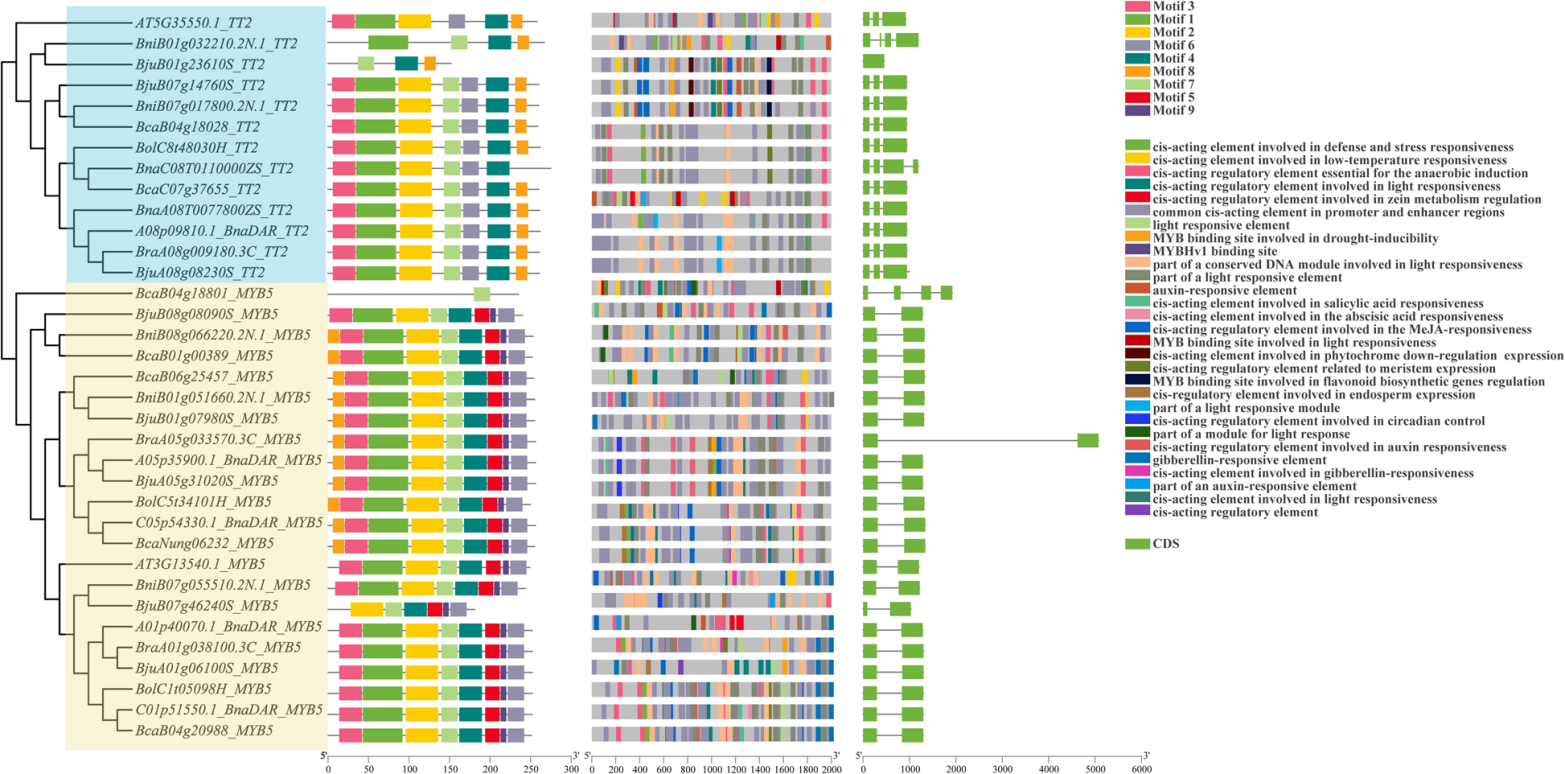
Phylogenetic, promoter characteristics, gene structure, conserved motifs analysis of *TT2* and *MYB5.* Predicted cis-elements in 7 co-differentially expressed R2R3-MYBs promoters and gene structure. Promoter sequences (-2,000 bp) of 7 co-differentially expressed R2R3-MYBs were analyzed using PlantCARE. Different shapes and colors represent different elements, PAFM was used for conserved domain prediction, and phylogenetic, promoter characteristics, gene structure, conserved motifs has been drawn by TBtools

**Fig. 6:**
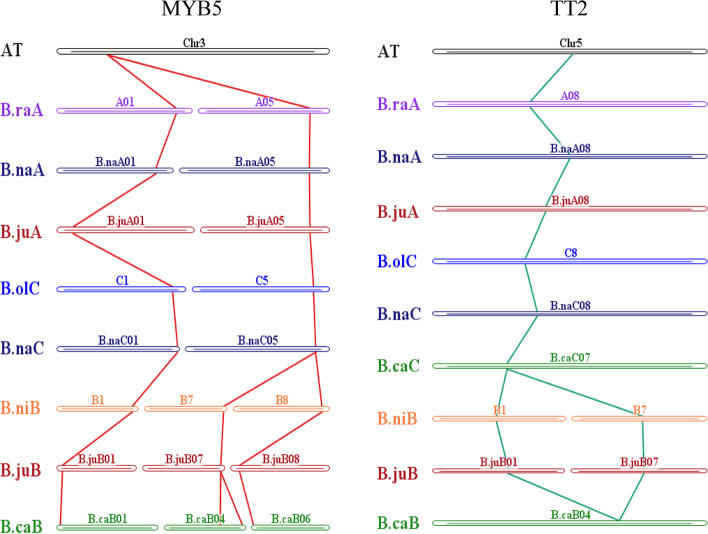
The distribution and collinearity of *MYB5* and *TT2* in A, B, C subgenome chromosomes

Phylogenetic analysis, gene structure analysis, and identification of conserved motifs contribute to a better understanding of gene functional variation and evolutionary pathways. To further explore the functions of *TT2* and *MYB5*, we analyzed their copy variation and chromosomal distribution, a total of 20 *MYB5* homologous copies and 11 *TT2* homologous copies were identified (Fig. 5). The 2,000 base pairs (bp) upstream of *TT2* and *MYB5* homologous of the coding region were used to predict *cis* regulatory elements via the PlantCARE online tool, the sequences of gene region were used to draw the gene structure, and protein sequences were used for phylogenetic and conserved domain analysis. (Fig. 5). Chromosomal mapping revealed two homologous copies of *MYB5* between the A and C subgenomes (except for *B. carinata*_(BcaC), and three between the B subgenomes, and with good correspondence between the subgenomes and chromosomes (Fig. 6). Similarly, *TT2* had one homologous copy between A and C subgenomes, and two copies between B subgenomes, and the correspondence between A, B and C subgenomes and chromosomes was good (Fig. 6).

Phylogenetic analysis of *TT2* showed that its distribution among A, B (B1, B7 chromosomes) and C subgenomes could be well clustered. The gene structure and conserved domains of different copies of *TT2* were basically the same, and only *BniB01g032210.2N.1* and *BjuB01g23610S* had large variation (Fig. 6). Since the number of copies of *MYB5* reached 25, its orthologous genes could be clustered well. Except for *BcaB04g18801* and *BjuB07g46240S*, the gene structure and protein conserved domain had less variation. At the same time, *BraA05g033570.3C* was found to have an insertion of about 4 Kb in the intron region. The number and types of regulatory elements in each anthocyanin-related gene were different, and the distribution of copies of regulatory elements with similar clusters was similar (Fig. 6).

### Expression pattern analysis of MYB5 and TT2 during seed coat development

**Fig. 7:**
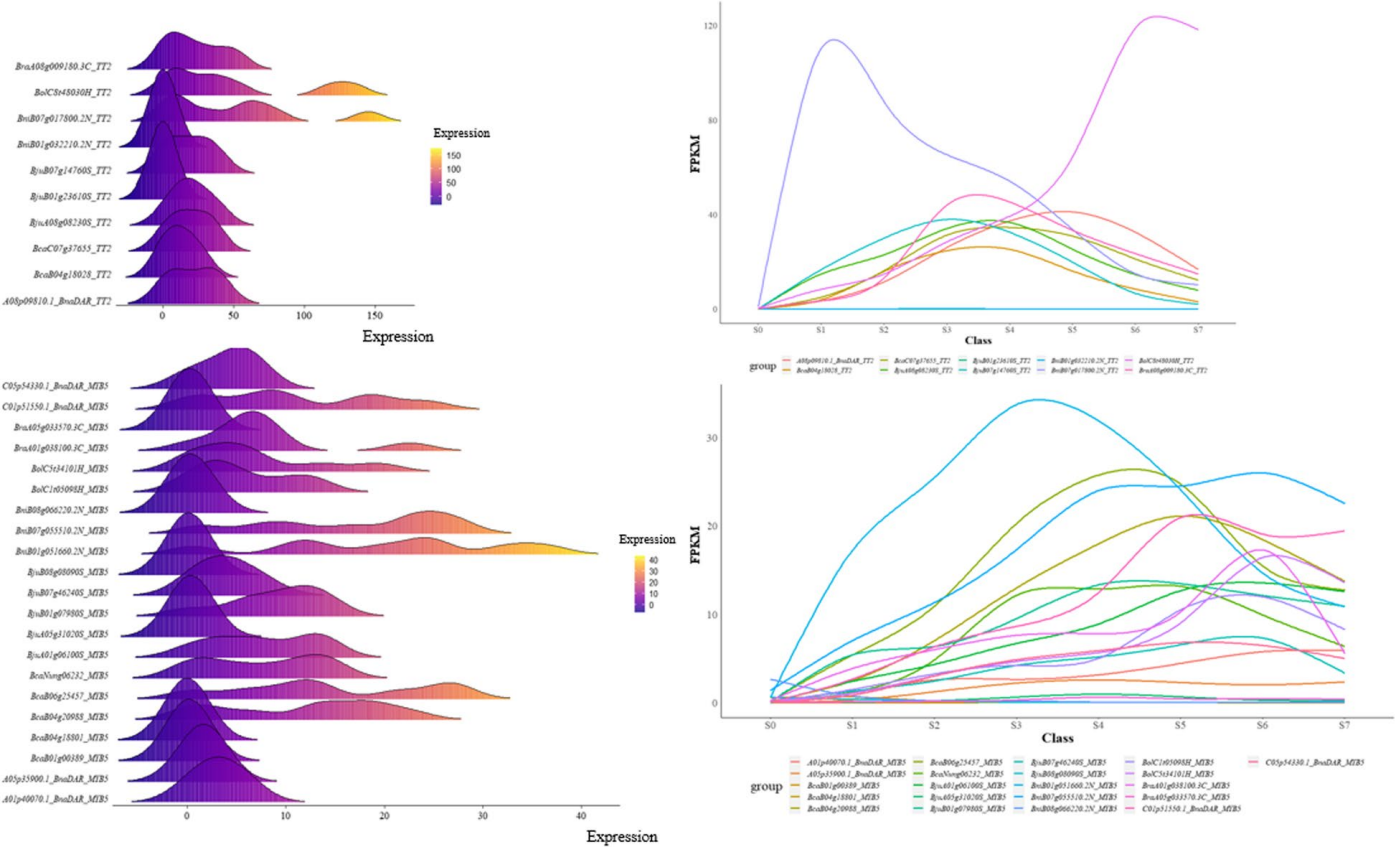
Expression pattern analysis of *MYB5* and *TT2* during seed coat development. **A** Expression trend of *TT2* homologous genes in six *Brassica* species; **B** *E*xpression curve of *TT2* homologous genes in six *Brassica* species; **C** *E*xpression trends of *MYB5* homologous genes in six *Brassica* species; **D** *E*xpression curve of *MYB5* homologous genes in six *Brassica* species

In order to gain insight into the expression patterns of *MYB5* and *TT2* during seed coat development of *Brassica* species, transcriptome data from eight developmental stages were selected for expression curve and trend analysis, respectively (Fig. 7). Most of the *MYB5* homologous gene copies initiated to be up-regulated in the S2 period, and the difference between the highest expression and the down-expression period was large. The most prominent one of *MYB5* homologous was *BniB01g051660.2N*, the expression level almost showed a parabolic trend from S1-S7 period, and the expression level was the highest in S3 period (Fig. 7). The homologous copy of *TT2* gene was mainly expressed at a high level in the S3-S4 period, *BniB07g017800.2N* had the highest expression level in the S1 period, and then decreased rapidly, while the *BolC8t48030H* expression was slowly up-regulated in the S0-S4 period, and expressed highest in the S6-S7 period (Fig. 7). In addition, six *MYB5* homologous copies (*BraA05g033570.3C*, *BniB08g066220.2N*, *BjuA05g31020S*, *A05p35900.1_BnaDAR*, *BcaB01g00389*, *BcaB04g18801*) and two *TT2* homologous copies (*BniB01g032210.2N*, *BjuB01g23610S*) were not expressed in eight seed coat development stages of six *Brassica* crops, and these genes mighty have been silenced during evolution (Supplementary Table S3).

## Discussion

The diploid *Brassica* genome underwent whole-genome triple duplication, followed by gene loss and reshuffling of genome fragments, which generated the currently grown tetraploid *Brassica* plants, followed by crossing, polyploidization, and breeding selection [43]. The diversity of allelic combinations among *Brassica* species may affect the expression and regulation of anthocyanin biosynthesis-related genes during seed coat development, resulting in variations in *Brassica* species seed coat color [13, 85]. Here, we finely identified the orthologous and paralogous genes of anthocyanin biosynthesis-related genes in six *Brassica* species, and determined their copy number and chromosomal distribution. Gene expression analysis revealed many important differences in anthocyanin metabolic pathways during seed coat development in *Brassica* species, especially the transcription factors *MYB5* and *TT2* were differentially expressed in eight developmental stages of six *Brassica* species, which provided new insights into the prediction and characterization of anthocyanin regulatory mechanisms and the evolutionary divergence that drove seed coat phenotypic differentiation in *Brassica* species.

### The anthocyanin-related genes in *Brassica* species

The genes related to the biosynthetic pathway of anthocyanins have been well identified, and the related gene functions have been reported in a variety of plants [10, 11, 41, 19]. In *Arabidopsis*, rice and maize, the biosynthesis, regulation and transport of anthocyanins, especially most of the catalytic enzymes and regulatory genes involved in anthocyanin synthesis, have been identified and functionally validated over the past few decades [9, 24, 25, 33, 35, 36, 42, 60, 76]. These studies have played an important role in comprehensively understanding plant anthocyanin biosynthetic pathways and regulatory mechanisms, and revealing the accumulation and metabolic characteristics of plant anthocyanins. In order to further promote the research on the transcriptional regulations of anthocyanins in *Brassica* crops, we have finely identified the anthocyanin-related genes of six species based on the deciphered genomic information. In this study, 1119 anthocyanin-related genes were identified in six *Brassica* species (Table 1), which gave the first report for *B. nigra*, *B. juncea* and *B. carinata*. In addition, the gene numbers of *B. rapa* (120), *B. oleracea* (131) and *B. napus* (258) were higher than those reported previously, 73 genes in *B. rapa* [14], 88 genes in *B. oleracea* [15], and 152 [16] and 157 [12] genes in *B. napus*. The main reasons were as follows: firstly, the reference genomes of *B. rapa*, *B. oleracea* and *B. napus* selected in this study were the latestly published and had the best completeness after genome-wide comparison,secondly, the recent v10 and v11 genome information of *Arabidopsis* was used and 55 anthocyanin-related genes were identified in *A. thaliana*,finally, different methods were used to identify orthologous and paralogous genes, which made the identification results more complete and accurate.

After a long period of natural evolution and human selection, *Brassica* crops in U's triangle have a lot of variation, including abundant types of leaf, stem, flower, silique and seed coat color variation. In the past two decades, from the mapping of the first gene related to anthocyanin synthesis in *B. rapa* [3] to the identification and characterization of a large number of genes related to anthocyanin synthesis, the transcriptional regulations of anthocyanin in *Brassica* species have been largely reported [3, 4, 17, 18, 64, 72, 73, 75, 77, 79, 82, 83]). However, because the three *Brassica* diploids underwent a whole genome triploidization (WGT) event and then crossed to form three allotetraploids, the copy number of anthocyanin-related genes varied, and the functional analysis of anthocyanin-related genes lagged due to the existence of multiple copies in *Brassica* species. This study found the copies of different anthocyanin-related genes varied, some copies lost in *Brassica* crops (e.g. *MYB11*), and some were massively expanded (e.g. *PAL*, *GST*, *AHA10*) (Table 1). Some copies of *MYB5* and *TT2* led to gene silencing due to structural variation during genome rearrangement (Figs. 5 and 7). Collectively, our findings provided an in-depth genome-wide view of genes involved in the anthocyanin biosynthesis pathways in six interconnected *Brassica* species, which further disclosed the formation mechanism of color variation in different tissues or organs of *Brassica* crops.

### Molecular mechanism of seed coat color variation in *Brassica* species

Yellow seed varieties of *B. rapa*, *B. juncea B. carinata* and *B. napus* have attracted the attentions of researchers due to their reduced thickness, reduced lignin content, and defects in PAs biosynthesis. The excellent characteristics of yellow seeds result in low seed coat pigmentation, high proportion of seed oil, and synthesis of crude protein [59, 38]. In this study, through genome-wide identification and combining transcriptome data from eight seed coat developmental stages of six *Brassica* species, we constructed anthocyanin-related expression profiles during seed coat development (Fig. 2). Structural genes related to anthocyanin synthesis pathway were significantly up-regulated during seed coat development, and only *MYB5* and *TT2* were differentially expressed in eight developmental stages of six *Brassica* species. In *Arabidopsis*, the *TT2-TT8-TTG1* complex played a major role in seed development, but three other MBW complexes (*MYB5-TT8-TTG1*, *TT2-EGL3-TTG1*, and *TT2-GL3-TTG1*) also functioning as tissue-specific ways were shown to be involved [76]. However, in *Brassica* species, Sharma and Mohapatra [63] first mapped the yellow seed coat color locus in *B. juncea* by constructing an RFLP linkage map. Subsequently, a number of markers for seed coat color of *B. rapa* [58], *B. juncea* [47], *B. carinata* [37] and *B. napus* [34] were developed, and the yellow seed coat trait was partially dominant and under monogenic control. In recent years, with the development of sequencing technology, the genome information of six *Brassica* crops has been deciphered successively, and the research on genes related to seed coat color has entered the stage of fine mapping, cloning and functional verification. *BjuA.TT8* in *B. juncea* [50], *BnaC.TT2.a* and *BnTT8* in *B. napus*  [84, 79]), *qSC9.1*, *qSCb9.1* and *qSC3.1* were successfully validated in *B. rapa* [81]. In *B. napus*, *BnaC.TT2.a* was reported to be associated with seed coat coloration, and its InDel at position 738 on exon 3 indicated changes in protein function significantly associated with seed coat color [84]. At the same time, transcriptome sequencing combined with metabolome analysis revealed new progress in the regulation mechanism of seed coat color and color substances. Structural genes and transcription factors such as *DFR*, *LDOX*(*ANS*), *BAN*, *TT3*, *TT8*, *TT18*, *TT10*, *TT12* and *TT19* were involved in the formation of seed coat coloration, and metabolites such as phenolic acids, epicatechin, flavonoids and PAs were important factors affecting seed coat formation in *Brassica* crops [55, 61, 64, 79].

Although the previous reports have provided a lot of information for revealing the variation of the seed coat color of *Brassica* species, the exact regulatory mechanism of the gene is less analyzed. In addition, there were few reports on the use of forward genetics to locate key genes of seed coat color and to analyze its molecular mechanism, which limited the use of molecular markers to assist in the breeding of yellow seed coat oilseed *Brassica*. Previous studies suggested that a single R2R3-MYB transcription factor combined with bHLH transcription factor and TTG1 protein to form the MBW transcriptional regulatory complex targeting *DFR*/*ANS* to drive anthocyanin synthesis and accumulation [76]. Our results further verified the important roles of *MYB5* and *TT2* and their homologous genes in the development of the seed coat of the six *Brassica* species, and from the analysis of the expression patterns and trends of different copies of *MYB5* and *TT2* at eight different developmental stages (Fig. 4). We speculate that during the formation of the MBW transcriptional regulatory complex, it is possible that multiple R2R3-MYBs (e.g. *MYB5* and *TT2*) associate simultaneously to form an MBW complex, which still needs to be verified by subsequent experiments. In summary, our results could broaden the scope of research on seed coat color and provided a reference for the subsequent improvement for yellow seed coat oilseed in *Brassica*.

## Supplementary Information

**Additional file 1.** Anthocyanin-related genes in B.carinata.

**Additional file 2.** Co-differentially expressed genes in six Brassica species seed coats eight development.

**Additional file 3.** Expression of TT2 in all eight seed coat development stages of six Brassica species.

## Data Availability

All materials and related data in this study are available upon request. If you need these materials and related data, you can download from Gene Expression Omnibus (https://www.ncbi.nlm.nih.gov/geo/) under accession no. GSE153257. If you need these materials and related data, you can contact Daoquan Xiang (daoquan.xiang@nrc-cnrc.gc.ca).

## Abbreviations

PAL: Phenylalanine ammonia-lyase
C4H: Cinnamate-4-hydroxylase
4CL: 4-Coumarate CoA ligase 4
CHS: Chalcone synthase
CHI: Chalcone isomerase
F3H: Flavanone 3-hydroxylase
FLS: Flavonol synthase
DFR: Dihydroflavonol 4-reductase
ANS: Anthocyanidin synthase
BAN: Anthocyanidin oxidoreductase
AT: Acyltransferase
GT: Glucosyltransferase
UFGT: UDP-flavonoid glucosyltransferase
TT2: TRANSPARENT TESTA 2
